# Experimentally induced antipredator responses are mediated by social and environmental factors

**DOI:** 10.1093/beheco/arz039

**Published:** 2019-04-12

**Authors:** Frank Groenewoud, Sjouke A Kingma, Kat Bebbington, David S Richardson, Jan Komdeur

**Affiliations:** 1 Behavioural Physiology and Ecology, Groningen Institute for Evolutionary Life Sciences, Faculty of Science and Engineering, University of Groningen, CC Groningen, the Netherlands; 2 Behavioural Ecology Group, Department of Animal Sciences, Wageningen University & Research,AH Wageningen, the Netherlands; 3 Centre for Ecology, Evolution and Conservation, School of Biological Sciences, University of East Anglia, Norwich, UK; 4 Nature Seychelles, Mahé, Republic of Seychelles

**Keywords:** antipredator defense, nest defense, nest predation, parental investment, Seychelles warbler, trade-off

## Abstract

Nest predation is a common cause of reproductive failure for many bird species, and various antipredator defense behaviors have evolved to reduce the risk of nest predation. However, trade-offs between current reproductive duties and future reproduction often limit the parent’s ability to respond to nest predation risk. Individual responses to experimentally increased nest predation risk can give insights into these trade-offs. Here, we investigate whether social and ecological factors affect individual responses to predation risk by experimentally manipulating the risk of nest predation using taxidermic mounts in the cooperative breeding Seychelles warbler (*Acrocephalus sechellensis*). Our results show that dominant females, but not males, alarm called more often when they confront a nest predator model alone than when they do so with a partner, and that individuals that confront a predator together attacked more than those that did so alone. Dominant males increased their antipredator defense by spending more time nest guarding after a presentation with a nest predator, compared with a nonpredator control, but no such effect was found for females, who did not increase the time spent incubating. In contrast to incubation by females, nest guarding responses by dominant males depended on the presence of other group members and food availability. These results suggest that while female investment in incubation is always high and not dependent on social and ecological conditions, males have a lower initial investment, which allows them to respond to sudden changes in nest predation risk.

## Teaser

Parental investment in antipredator defense depends on its relative costs and benefits. By experimentally manipulating nest predation risk in the Seychelles warbler, we show that sex-specific antipredator defenses are modified by social and ecological factors. Females show the highest investment in the nest, while males invest less, enabling them to increase their investment when predation risk increases.

## INTRODUCTION

Predation risk is an important factor explaining variation in life history and behavior in many animals (Barbosa and Castellanos 2005; Caro 2005; Creel and Christianson 2008). In birds, nest predation is one of the most common causes of nest failure and is, therefore, one of the key drivers in the evolution of avian breeding biology (Ricklefs 1969; Martin 1995). For example, individuals can vary nest site location or clutch size according to predation risk (e.g., Martin 1995; Eggers et al. 2006; Dillon et al. 2018), and parents might visit the nest less often when nest predation threat is high (e.g., Ghalambor and Martin 1999; Fontaine and Martin 2006; Ghalambor et al. 2013). If antipredator behavior is costly, then individuals experiencing different levels of nest predation risk should adjust such behavior accordingly (Lima 2009). However, investment is often constrained by trade-offs between current and future reproduction, or between current reproduction and other important activities (Trivers 1972; Stearns 1989; reviewed in Martin 1995). Experimental studies are necessary to determine which conditions shape antipredator responses, and the trade-offs underlying antipredator responses, but such studies are scarce (Lima 2009). Here, we experimentally increased nest predation risk in a cooperatively breeding passerine to provide insights into whether and how social and environmental factors shape antipredator responses.

Increased nest attendance or vigilance is a common response to increased nest predation risk and can improve predator detection (Montgomerie and Weatherhead 1988; Caro 2005) and nesting success (Komdeur and Kats 1999). Such behavior is also hypothesized to be costly, because individuals are unable to simultaneously invest in other activities, such as foraging (Komdeur et al. 1999). If so, individuals in areas with high food availability may suffer fewer costs of nest defense compared to individuals from lower quality areas (Duncan Rastogi et al. 2006). Similarly, the costs and benefits of increased antipredator behavior can also differ between males and females (Montgomerie and Weatherhead 1988). For instance, males, who are larger in many passerine species (Ranta et al. 1994; Mills 2008), may engage more in risky defense against predators than females if they are more effective and/or have a lower risk of injury (Andersson and Norberg 1981). Thus, sex differences, and variation in environmental conditions can alter the costs and benefits of antipredator behavior, and shape the trade-offs between investment in current and future reproduction.

Predator defense strategies may also depend on the social context (Clutton‐Brock 1991). For example, if individuals can confront a predator together, they may mount a more effective and less risky defense than those who defend the nest alone (Weatherhead 1989); such joint-defense is likely a major benefit of group living (Krause and Ruxton 2002). Additionally, investment by other individuals might allow a focal individual to reduce its own investment (i.e., load lightening; Johnstone 2011). However, this is not always the case (Hatchwell 1999; Valencia et al. 2006), and the effects of the social environment on the expression of individual antipredator behaviors are complex and generally not well understood.

In the facultative cooperatively breeding Seychelles warbler (*Acrocephalus sechellensis*) egg predation is the primary cause of nest failure (Komdeur and Kats 1999). The main nest predator is the Seychelles fody (*Foudia sechellarum*; hereafter “fody”), a weaver bird that takes eggs from unattended warbler nests (Komdeur and Kats 1999). Seychelles warblers on Cousin Island typically lay single egg clutches (91% of clutches; Bebbington et al. 2017), which means that a predation event in most cases renders the entire breeding attempt unsuccessful (Komdeur 1996). In response to nest predation, warblers have evolved direct (attacks and alarms) and indirect (nest guarding) antipredator behaviors (Komdeur 1991; Veen et al. 2000).

The species is well-suited to test how antipredator behavior varies with social and environmental factors: Seychelles warblers feed exclusively on arthropods, and arthropod availability is variable across the island (Komdeur and Daan 2005). Local food availability may, therefore, play an important role in modulating the expression of antipredator behaviors. Furthermore, several social components of the Seychelles warbler system might be central in driving antipredator behaviors. First, nest defense tactics are sex-specific: males engage in nest guarding (showing vigilance behavior close to the nest; Slack 1976), whereas females incubate and prevent fodies from accessing the eggs. Both behaviors reduce the likelihood of nest predation (Komdeur 1991; Komdeur and Kats 1999). Second, males mostly guard the nest when females leave the nest, but there is some overlap between nest guarding and incubation, particularly at the beginning and end of female incubation bouts. Predators can, therefore, be confronted by either the male alone, the female alone, or both parents together, and variation in antipredator responses can indicate different costs of nest defense due to the social environment. Third, dominants can be accompanied by 0–4 subordinates (Komdeur 1991; Kingma et al. 2016), which can help with incubation (females only; Komdeur 1991, 1994). However, the role of subordinate Seychelles warblers in mitigating predation risk is unknown.

Here, we experimentally increased the perceived risk of egg predation in Seychelles warblers by using a mounted fody model with call playback to simulate an imminent threat at the nest. We assessed the direct antipredator responses of individuals to these models (attacks and alarm calls), as well as the subsequent changes in indirect antipredator behavior (incubation and nest guarding behavior) and compared these to a presentation with a nonpredator control model. We test 3 hypotheses: 1) Parents increase antipredator behaviors (nest guarding or incubation) in response to experimentally increased nest predation risk, depending on the availability of food or parental sex, 2) Parents respond differently to a direct predator threat depending on whether they confront a predator alone or together, 3) Subordinates contribute towards nest defense and their presence affects the dominant birds’ antipredator behavior. Our results shed light on how group members engage in different types of nest defense behaviors and how these behaviors are affected by social and environmental contexts.

## MATERIALS AND METHODS

### Study population

The Seychelles warbler is a small cooperatively breeding passerine endemic to several islands in the Seychelles. The main study island of Cousin (ca. 29 ha; 4°19′53.6″S 55°39′43.3″E) is saturated with Seychelles warbler territories, and the population is stable around 320 adult birds (Brouwer et al. 2009). Since 1997 nearly all birds (97%) on the island are individually identifiable by a unique combination of color rings and a metal ring (Hammers et al. 2015; Komdeur et al. 2016). The sex of all ringed individuals was confirmed by molecular sexing (Richardson et al. 2001). To find nests, dominant females in each territory were followed for at least 15 min every 3–4 days. To determine the date of egg laying, we checked all nests at least every fourth day before nest completion and every other day after.

Seychelles warblers feed exclusively on arthropods (Komdeur 1991), and territory quality can, therefore, be estimated as the availability of arthropod prey, according to the methods in Komdeur (1992) and Brouwer et al. (2006). Briefly, we counted the number of arthropods on the underside of 50 leaves of all main tree species at 13 different locations, representative of each part of the island. We then estimated the cover of each of these tree species at different strata of the canopy for each territory. Arthropod counts per tree species were then multiplied by the cover of each tree species for each territory. The resulting measure of territory quality (i.e., arthropod density) was log transformed and mean centered.

### Nest predator presentation

Predator presentation experiments were performed on Cousin Island between the 19th of July and the 2nd of September 2015, between 10 and 12 AM or 2 and 5 PM. The Seychelles fody is listed as “near-threatened” on the IUCN red list (BirdLife International 2013), so we were unable to obtain a taxidermic model of this species. Instead, we used a mounted female house sparrow (*Passer domesticus*), which is very similar to the Seychelles fody in size and appearance. An earlier investigation into predator recognition in Seychelles warblers showed no differences in antipredator responses between a caged Seychelles fody and a caged mounted female house sparrow (Veen et al. 2000). Two different mounted house sparrows were used to increase generalizability (Johnson and Freeberg 2016). Like Veen et al. (2000), we used a mounted barred ground dove (*Geopelia striata*), which occurs naturally on Cousin, as a nonpredator control. Using an 8-m-long fiberglass telescopic pole, we presented either a mounted house sparrow (*N* = 19) or barred ground dove (*N* = 11) ~1 m from the Seychelles warbler nest during incubation. Practical constraints meant that experiments were performed during different stages of nest incubation (mean number of days after onset of incubation = 8.9, range = 3–15 days). All experiments were performed on different nests in different territories apart from one territory, where we used 2 different predator models for 2 consecutive breeding attempts. During the presentation, we played calls of the presented species (from the Xeno-canto bird sound database: www.xeno-canto.org) using a speaker placed 5–10 m from the nest. Audio playbacks were standardized by removing background noise and repeating 2 call bouts every 30 s for the full length of the presentation (Supplementary Information). We used different recordings for each mounted house sparrow model.

We recorded the number of attacks—pecking and dive bombing (i.e., rapidly flying overhead and pecking at the model in flight)—and alarm calls by the dominant male, dominant female, and subordinates of either sex (when present) using a GoPro (Hero 3+) mounted on the telescopic pole, 1 m from the model. We used a voice recorder, in addition to the video recordings, to record the identity and behavior of birds during the experiment. These recordings were later processed and the number of alarm calls and attacks was quantified using the software BORIS (Friard and Gamba 2016). The presentation ended 5 min after the arrival of the first individual at the nest area, visible to the observer.

Nest guarding was defined as individuals perching <2.5 m from the nest while no female was on the nest (Komdeur and Kats 1999). To assess whether individuals showed more nest guarding (males) or incubation behavior (females) after an encounter with the simulated nest predator, 1 of 3 different observers recorded the behaviors of the group individuals for 1 h both before and after the presentation of the mounted bird. During the second observation—which started 5 min after the end of the predator or control presentation—we used the same playback to simulate the continued presence of the predator or control bird in the territory. In all but 2 cases, observations before and after the presentation were conducted by different observers.

## STATISTICAL ANALYSES

### Attacks and alarms

Attacks toward the nonpredator model (dove) were rare: in all 11 nonpredator presentations, only 3 individuals (in 3 different territories) attacked the model. Therefore, in the analysis of attacks, we focused on responses toward the predator model only, while analyses of alarm calls also included the nonpredator model. We fitted either number of alarms, or attacks, as the response variable in separate generalized linear mixed models assuming a Poisson error. We fitted an individuals’ *status* (dominant male, dominant female, or subordinate) and the *presentation type* (fody vs. dove; for alarms only) as predictors. To determine whether the number of alarm calls or attacks differed when individuals were alone or together, we also included whether other defenders were present as a binary variable. Individuals that “arrived together” were either those that joined a partner that was already present, or that arrived with another individual, within 10 s of each other. We included the log-transformed time spent alone or together (in min) as an offset in both models, to account for the time individuals spent either alone or together during the presentation. We also analyzed whether there were differences between the 2 different predator models, by including this as covariate in our models, and we included *territory* as a random effect to account for the nonindependence of observations within territories. We included *embryo age* (i.e., the number of days after the onset of incubation) to account for potential differences in the motivational state in the breeding cycle. Following Veen et al. (2000), we only analyzed the behaviors during the first 2 min of observations for each individual after arrival.

### Changes in incubation and nest guarding

To investigate whether individuals increased nest guarding (dominant males) or incubation (dominant females) after being confronted with a nest predator, we analyzed these behaviors separately using linear mixed models with varying intercepts for each territory. We included the interaction between *presentation type* (predator or nonpredator) and *observation time* (before or after the presentation) to test whether the change in behavior before and after the presentation was different for nests exposed to predator and control models. We also tested whether changes in dominant behavior were affected by arthropod density and incubating subordinate presence by including interactions between these 2 variables and *observation time.* To account for the possibility that incubation and nest guarding behavior varies with embryo age, we included *embryo age* in both analyses and tested for an interaction between *embryo age* and *observation time*. We allowed for random intercepts between observers to account for between-observer variation.

We used a model selection approach based on the Akaike information criterion (Akaike 1973) with small sample size correction (AICc; Hurvich and Tsai 1989). We fitted full models and dropped variables (starting with interactions) if doing so led to a lower AICc value (Burnham and Anderson 2002; Burnham et al. 2010), as assessed in the package *AICcmodavg* (Mazerolle 2013). Variables that were central to the testing of our hypotheses (i.e., *presentation type, observation time*, and *status*) were not removed. Removed variables and interactions were reentered for estimation of their effects using likelihood ratio tests (LRT) on nested models assuming a χ^2^ distribution. All models were fitted using the package *lme4* (Bates et al. 2015). We used package *multcomp* (Hothorn et al. 2008) to test whether slope estimates contained in higher level interactions differed significantly from zero. Effect sizes (β) are reported as means ± standard errors.

## RESULTS

### Alarm calls and attacks to model presentation

**Figure 1:**
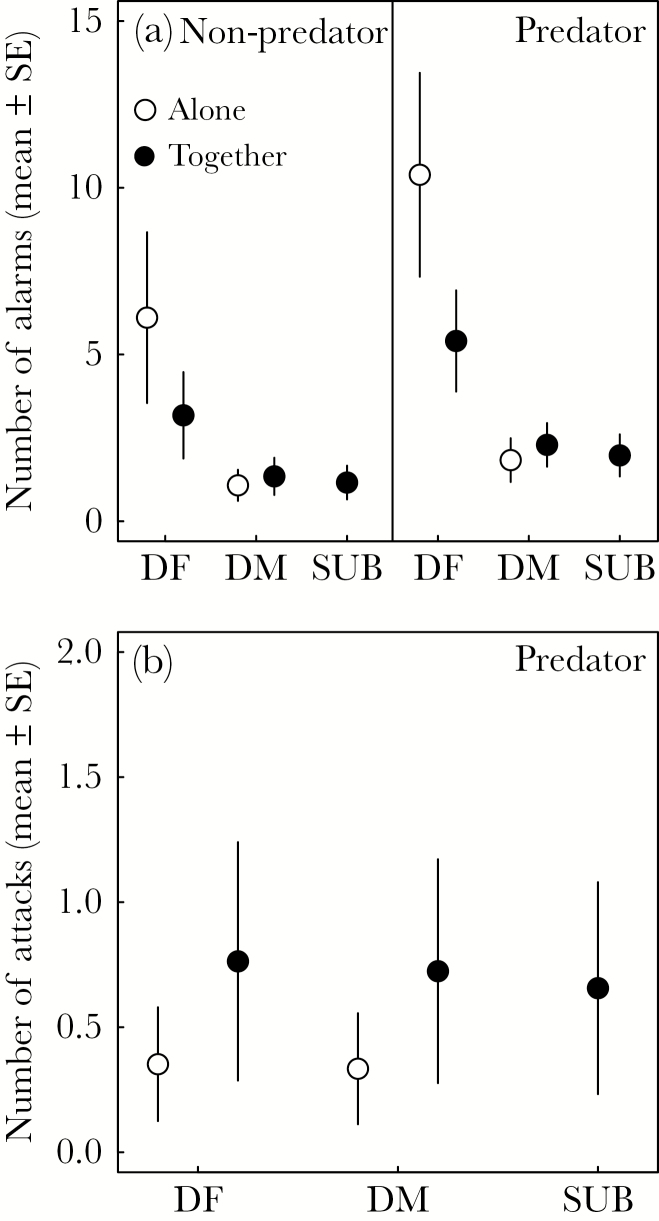
The mean model predicted (±SE) number of alarm calls (a) and attacks (b) per minute for Seychelles warblers when they were alone (open circles) or together (filled circles) during an experimental presentation of a nest predator (*N* = 19) or nonpredator (*N* = 11). DF = dominant female, DM = dominant male, SUB = subordinate.

All predator presentations evoked alarm calls from at least one individual in the territory (19/19), while this was true for only 4/11 (36.3%) of nonpredator presentations. This pattern was similar for attacks, where 12/19 (63.3%) predator presentations lead to an attack of the mounted model, but only 3/11 (27.3%) nonpredator presentations did so. Individuals did not alarm more during the predator presentation than during the nonpredator presentation (β = 0.53 ± 0.49, $\chi_{1}^{2}$ = 1.19, *P* = 0.28; Figure 1a), and there were no differences between the 2 predator models used (β = −0.15 ± 0.54, $\chi_{1}^{2}$ = 0.07, *P* = 0.79). Dominant females alarm called more than dominant males (β = −1.01 ± 0.10, *z* = −10.53, *P* < 0.001; Figure 1a) and subordinates (β = −1.05 ± 0.17, *z* = −6.10, *P* < .001; Figure 1a), but dominant males and subordinates called at similar rates (β = −0.03 ± 0.18, *z* = −0.16, *P* = 0.99; Figure 1a). Dominant females alarm called more when they were alone than when they were together, but no such effect was present for dominant males, who did not adjust their alarm calling rate according to whether they were alone or together (interaction β = 0.88 ± 0.32, $\chi_{1}^{2}$ = 8.03, *P* = 0.005; Figure 1a). The number of alarms was independent of embryo age (β = −0.08 ± 0.05, $\chi_{1}^{2}$ = 2.16, *P* = 0.14). Interestingly, the 5 subordinates that participated in attacks or alarms (of the 6 that were seen during the presentations) always arrived after the dominant female or dominant male: therefore, differences in attack or alarm rates depending on whether these subordinates confronted the model together or alone could not be estimated.

There was no difference in the number of attacks between dominant females, dominant males or subordinates ($\chi_{2}^{2}$ = 0.60, *P* = 0.74). Individuals attacked the predator model more often when they were together than when they were alone (β = 0.79 ± 0.27, $\chi_{1}^{2}$ = 9.74, *P* < 0.01; Figure 1b), and, unlike for alarm calling, this did not differ between dominant females and dominant males (interaction β = 0.63 ± 0.87, $\chi_{1}^{2}$ = 0.59, *P* = 0.44). Individuals tended to attack one of the predator models more than the other (β = 1.59 ± 0.83, $\chi_{1}^{2}$ = 3.49, *P* = 0.06). Attack rates decreased with embryo age (β = −0.29 ± 0.10, $\chi_{1}^{2}$ = 8.23, *P* < 0.01).

### Changes in nest guarding and incubation

**Figure 2:**
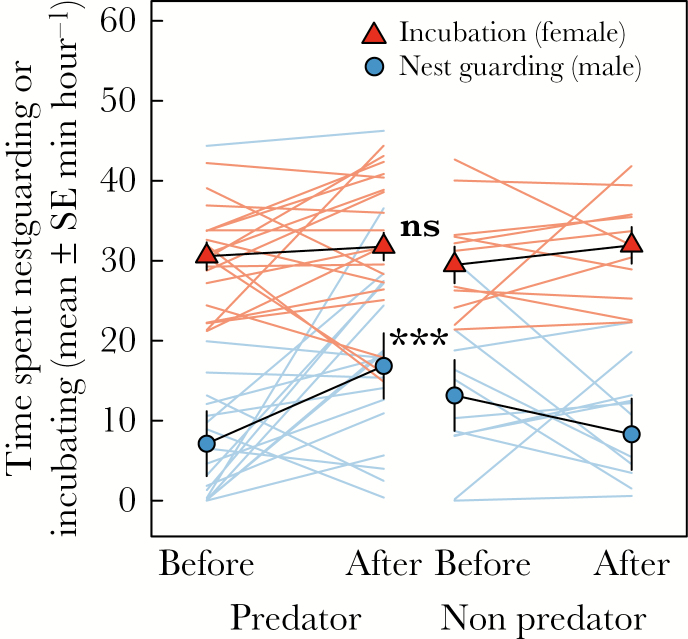
Time spent nest guarding or incubating by Seychelles warbler dominant males (blue circles) and dominant females (red triangles), respectively, before and after a nest predator (*N* = 19) or nonpredator (*N* = 11) presentation. Black lines represent mean predicted responses with standard errors, while colored lines show individual changes in behavior. Significance indicators reflect whether slopes differ from each other (***P < 0.001) or not (NS).

**Figure 3:**
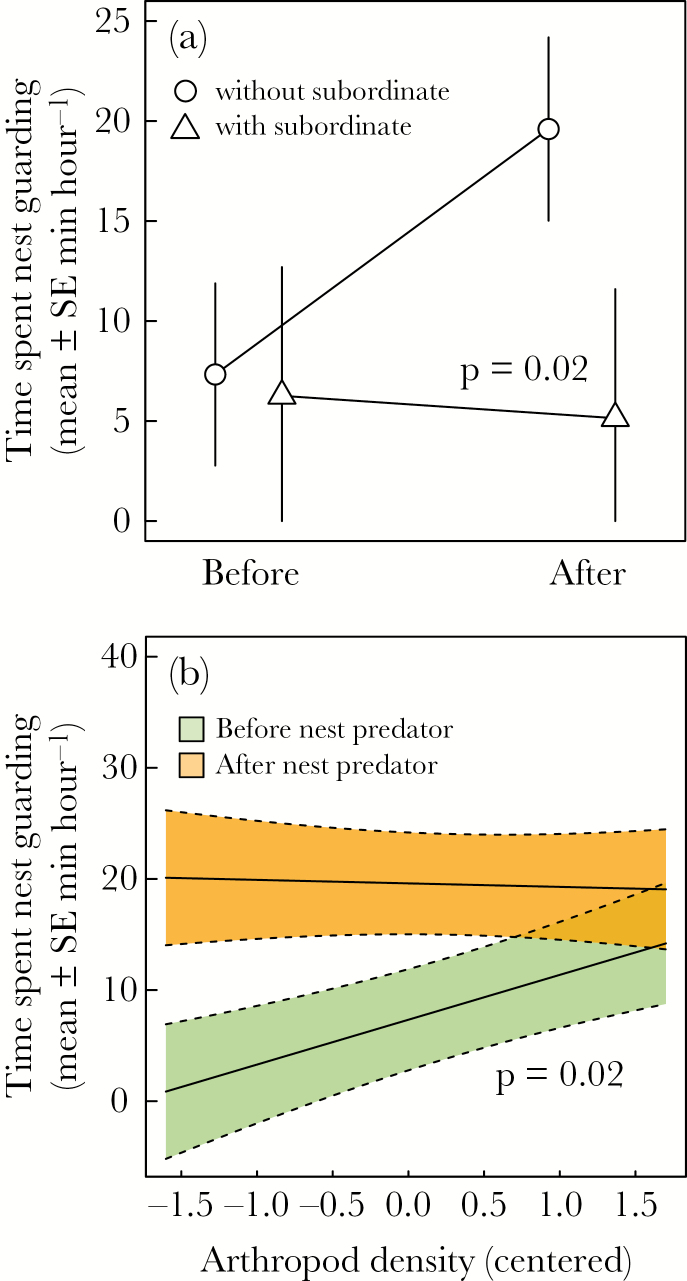
Changes in time spent nest guarding by dominant male Seychelles warblers as a result of an experimental presentation with a nest predator in relation to (a) having an incubating subordinate present in the territory (*N* = 4) or not (*N* = 15), and (b) food availability. *P* values relate to the hypothesis that slopes differ from each other.

Dominant females spent most of their time incubating (97% vs. 3% nest guarding), while dominant males almost exclusively nest guarded (99.2%). Subordinates (4 females) showed a mixed investment (82.4% incubation vs. 17.6% nest guarding), while one subordinate male nest guarded for 84 s, but showed no alarms or attacks during the presentation. Dominant males increased their nest guarding duration significantly after the predator presentation (β = 0.21 ± 0.04, *z* = 5.47, *P* < 0.001), while males nest guarded less after the nonpredator presentation (β = −0.09 ± 0.04, *z* = −2.03, *P* = 0.04). Consequently, dominant males increased nest guarding more after a nest predator presentation than after a nonpredator presentation (interaction β = 0.29 ± 0.06, $\chi_{1}^{2}$ = 19.79, *P* < 0.001; Figure 2). Although the number of territories with incubating subordinates was low (*n* = 4), dominant males did not change nest guarding after the experiment when there was an incubating subordinate present in the territory, compared to territories where no subordinate was present (interaction β = −0.22 ± 0.09, $\chi_{1}^{2}$ = 5.97, *P* = 0.02; Figure 3a). Consistent with previous results (Komdeur and Kats 1999), nest guarding by dominant males before the predator presentation was higher in territories with higher arthropod density. However, time spent nest guarding was independent of arthropod density after the predator presentation (interaction β = −0.07 ± 0.03, $\chi_{1}^{2}$ = 5.21, *P* = 0.02; Figure 3b). Changes in male nest guarding duration (interaction β = <0.01 ± 0.01, $\chi_{1}^{2}$ = 1.22, *P* = 0.27), and the total time males spent nest guarding (β = <0.01 ± <0.01, $\chi_{1}^{2}$ = 0.50, *P* = 0.48) were independent of embryo age.

Dominant females did not change their incubation behavior in response to either the predator presentation (β = 0.02 ± 0.03, *z* = 0.58, *P* = 0.56) or the nonpredator presentation (β = 0.04 ± 0.05, *z* = 0.89, *P* = 0.37). Consequently, changes in dominant female incubation behavior were independent of the type of model presented (interaction β = −0.02 ± 0.06, $\chi_{1}^{2}$ = 0.14, *P* = 0.71; Figure 2) There was a nonsignificant tendency for dominant females to incubate less when there was an incubating subordinate present (β = −0.11 ± 0.06, $\chi_{1}^{2}$ = 2.96, *P* = 0.09), but change in incubation duration after model presentations was not dependent on whether there was an incubating subordinate present (interaction β = 0.06 ± 0.10, $\chi_{1}^{2}$ = 0.51, *P* = 0.48). Changes in dominant female incubation behavior tended to be higher in territories with higher arthropod density (although not significantly so; interaction β = 0.06 ± 0.03, $\chi_{1}^{2}$ = 3.24, *P* = 0.07). This was due to a marginally significant negative relationship between arthropod density and incubation duration before the model presentations (β = −0.11 ± 0.06, *z* = −2.00, *P* = 0.05), while there was a positive (but not significant) relationship between arthropod density and the duration of incubation after the model presentations (β = 0.10 ± 0.06, *z* = 1.60, *P* = 0.11). Changes in female incubation duration (interaction β = <0.01 ± 0.01, $\chi_{1}^{2}$ = 0.01, *P* = 0.91), and the total time females spent incubating (β = <0.01 ± <0.01, $\chi_{1}^{2}$ = 0.63, *P* = 0.43) were independent of embryo age.

## DISCUSSION

### Sex-based differences in predator response

In contrast to Veen et al. (2000), who found that males had higher attack rates than females, we found no such difference, but we did find that females alarm called more than males overall. Dominant males increased their nest guarding behavior after our nest predator presentations, but no such change was found in terms of incubation behavior by dominant females. The lack of response in females is likely the result of the higher overall female investment and the trade-off between incubation (which is likely to be energetically more demanding than nest guarding) and time spent foraging (Reid et al. 2002; Tinbergen and Williams 2002). On average females incubate ca. 50% of their time, while males do not incubate and only spent 17% of their time on nest guarding (only pre-experiment nest watches), leaving males with more opportunity to respond to the increased threat, compared with females. Additionally, relatively low incubation effort and the unwillingness by females to further increase incubation effort could be expected in a long-lived tropical species where future reproduction is often favored over increased risk and expenditure in the current breeding attempt (Martin et al. 2015).

### Predator responses in a social context

In species where more than one individual provides parental care, individuals might alter their antipredator responses depending on the social context (Chase 1980; Clutton‐Brock 1991). Interestingly, Seychelles warblers attack a nest predator model more often when they are together than when they are alone (Figure 1b). Similar patterns have been found in other species, for example, great tits *Parus major* (Regelmann and Curio 1986) and African female lions where some, but not all, individuals showed cooperative strategies that were conditional on the social context (Heinsohn and Packer 1995). It is likely that individuals are more likely to attack together because of the benefits of additional vigilance by others. Alternatively, individuals might be signaling a willingness to invest in the current brood as a display of quality (Zahavi 1975; Fuong et al. 2015), or as part of parental negotiations over care, in the hope that their partner will also increase investment (Johnstone and Hinde 2006; Johnstone et al. 2013).

Dominant females alarm called more when they were alone than when they were together, which is consistent with at least 2 functions that have been ascribed to alarm calls in other species: 1) when alarm calls function to signal to the predator that it has been seen, but attacking alone is too risky (e.g., Zuberbühler et al. 1997), and 2) to signal the presence of a threat to other group members (reviewed in Caro 2005). Interestingly, dominant males did not increase alarm rates if they confronted the model presentation alone. Dominant males generally alarm called less than females and did not compensate for this by showing more attacks than dominant females. Our results, therefore, suggest that dominant Seychelles warbler females show more risk-averse antipredator behaviors when they are alone, switching to more direct aggression when they confront a nest predator when they have social support. The benefits of social support for females by the presence of their male was also found experimentally in another context, where male presence during foraging reduced female vigilance, resulting in improved foraging ability and time spent incubating eggs (Fedy and Martin 2009).

Subordinates did not always participate in nest defense, neither by the direct defense (alarms and attacks) toward a predator nor by incubation or nest guarding. It is possible that subordinate nest defense strategies are conditional (e.g., perhaps based on relatedness or body condition as observed for provisioning in this species; Richardson et al. 2003; van de Crommenacker et al. 2011) as they are less consistent than that of the breeders. This is further illustrated by the fact that even when subordinates participated in nest predator defense, they always arrived after the dominant female or dominant male. However, when they did participate in defense, they alarm called as often as dominant males, and attack rates were similar to dominant males and females (Figure 1b). Although we conducted our experiment in only 4 territories with incubating subordinates, our results suggest that males can also benefit from the presence of incubating subordinates: males without incubating subordinates showed a much stronger response after the simulated nest predation threat, while such an effect was smaller and not significant for females (Figure 3a). Load lightening is a common benefit of subordinate help, and observed in many cooperative breeders (e.g., Hatchwell 1999), including the Seychelles warbler (Komdeur 1994).

### Predator responses and embryo age

Surprisingly, we found that attacks toward the nest predator model decreased when eggs had been incubated for more days, which is counter to the general hypothesis that nest defense should increase with the increased reproductive value of the clutch or reduced nesting potential (Montgomerie and Weatherhead 1988). However, results for this hypothesis have been mixed, with some species showing no change in nest defense behavior as the brood ages, and others showing decreased investment, similar to our results (reviewed in Caro 2005). Several explanations exist for the decline of antipredator responses with embryo age. For instance, parents could experience a decrease in body condition as the brood ages due to investment in incubation and nest guarding. Additionally, testosterone levels suggested to drive aggression and nest defense (Collis and Borgia 1992), might decrease with advancing embryo age, as has been shown, for instance, in female canaries *Serinus canaria* (Schwabl 1996).

### Predator responses and food availability

Nest guarding responses by dominant males were dependent on arthropod density: time spent nest guarding in high-quality territories was already high and did not change much as a result of our model presentations, while dominant males in low-quality territories showed a significant increase in nest guarding (Figure 3b). This result is in line with a previous study in the Seychelles warbler that showed a similar correlation between male nest guarding investment and territory quality (Komdeur and Kats 1999). Our results thus indicate that male nest guarding behavior can be temporally increased when the risk of nest predation is high, but that low food availability may prevent dominant males from keeping up high levels of close nest guarding over a longer period of time (Martin 1995). Interestingly, where Komdeur and Kats (1999) found no relationship between territory quality and female incubation, we found that dominant females tended to show a decrease in incubation duration with increasing territory quality, which was similar in strength to the increase in nest guarding behavior for dominant males. Whether part of female incubation behavior is compensatory and functions to protect the clutch is an interesting question, but male removal experiments would be necessary to show this conclusively. Fedy and Martin (2009) removed males of 2 species of songbird, who guard females during foraging, and found that male removal increased female vigilance with negative effects on female foraging efficiency and incubation attendance. Nest guarding in the Seychelles warbler could have a similar function and allow females to forage further away from the nest in areas of high food availability. The main difference between Komdeur and Kats (1999) and this study is that their measure of territory quality included territory size (i.e., is a measure of total arthropod abundance), while our study did not (i.e., measures arthropod density). The latter could be a better reflection of female foraging efficiency during incubation off bouts and, therefore, of the trade-off between incubation and territory quality.

### Conclusions

Our results show different responses to short-term increased nest predation risk between different group members in the Seychelles warbler. We highlight the fact that male vigilance is likely much more flexible perhaps because 1) it is unconstrained by thermal requirements to the egg(s) and 2) the initial investment is much lower than that of females, leaving more opportunity for males to respond to increased risk. This is further illustrated by the finding that male nest guarding behavior is conditional on arthropod density and on subordinate help, suggesting that this behavior is costly, and that these costs can be alleviated under favorable food availability and social conditions. Together, these results show that antipredator behavior can differ substantially according to individual, social, and environmental conditions.

## FUNDING

This work was supported by Netherlands Organisation for Scientific Research (NWO) TOP grant (854.11.003 to J.K.), ALW grant (823.01.014 to J.K.), Veni fellowship (863.13.017 to S.A.K.) and Natural Environment Research Council (NERC) grant (NE/K005502/1 to D.S.R.).

## Supplementary Material

arz039_suppl_Supplementary_Dove-Playback-1

arz039_suppl_Supplementary_Fody-Playback-1

arz039_suppl_Supplementary_Fody-Playback-2
